# Healthcare seeking – who, when and why?

**DOI:** 10.1080/02813432.2024.2407878

**Published:** 2024-09-23

**Authors:** Peter Haastrup, Linda Huibers

**Affiliations:** a Research Unit of General Practice, Department of Public Health, University of Southern Denmark, Odense, Denmark; b Research Unit for General Practice, Aarhus, Denmark

A Monday morning with the waiting room being full, one of Peter’s patients said with a smile: “It seems you don’t have to worry about advertising; just open the door and you have a full schedule.” The same appears to be the case in the out-of-hours service; just log on to the system and the phone starts ringing.

Although we will probably never run out of work as general practitioners (GPs) and we focus our full attention on the patients we see each day, we do sometimes ask ourselves: “Why did *this* patient need to see me *today* and who did I not see today that could have benefitted from my attention?”. Despite the Scandinavian healthcare systems implying free and equal access to general practice, the process of deciding to seek medical attention can be much more complex than at first sight.

We are trained to ask the patients in front of us why they came exactly today, with this problem, to our practice. Often worrying about a symptom or the symptom’s negative effect on daily activities can be important factors in the process of deciding to contact the GP [1]. But patients often consider a lot of factors when deciding whether to seek help and symptom responsiveness is not only a cognitive process of bodily sensations becoming a symptom but a more complex interplay between biological, psychological, social, and cultural dimensions as well [2]. Further, health beliefs of the patients, previous experiences, and the organization of healthcare systems are important in the decision-making process of healthcare seeking [3, 4]. Although, as healthcare professionals, we want patients to seek care timely to avoid delay of potentially serious diseases, we simultaneously warn them not to contact healthcare too quickly as to limit demands and overcrowding [5]. So while healthcare seeking campaigns have been carried out, especially focusing on early recognition of stroke symptoms, myocardial infarction, or potential cancer symptoms [6], other regional initiatives have focused on reducing the need of contacting the GP or out-of-hours care, for example in case of fever [7]. This ambivalence entails a risk of confusing the citizens while trying to provide information and education.

Not only patients struggle to assess the timeliness of healthcare seeking; also healthcare professionals find this challenging. Too quick healthcare seeking can use healthcare resources unnecessarily, puts pressure on the system, and limits time for more medically relevant issues. Also, there is a risk of medicalization, as by having a healthcare contact for a harmless self-limiting symptom, a patient can receive diagnostics and treatment, which could include overdiagnosis and overtreatment and could give an incentive to come again for similar issues. Too late seeking can delay treatment of serious conditions such as cancer or cardiovascular disease, and seriously affect treatment and prognosis. Therefore, the balance between not too early and not too late is often debated. Unfortunately, the discussions surrounding timely healthcare seeking are often somewhat polarized in favor of either increased early diagnosis or concerns about risk of overdiagnosis or medicalization, which often leaves little space for the complex reality full of nuances and contradictories.

It is important to keep in mind that a significant process of considerations often precedes the patients’ contacting us and many symptoms are dealt with without contacting the healthcare system [8]. Even for potential cancer symptoms that from a medical perspective warrant investigation, we only see the top of the iceberg in our consultation rooms. One of the factors that is associated with less contacts to the GP when experiencing lung cancer symptoms is smoking. Although smokers constitute the main risk group for lung cancer and often have pulmonary symptoms, their odds of contacting their GP with symptoms are much lower than non-smokers experiencing the same symptoms [9]. Yet, smokers are often worried about what the doctor may find. It is also thought-provoking that the smokers frequently are too embarrassed to contact the GP. Have we come so far in our strive for health promotion and a normative disease preventing lifestyle that patients with certain lifestyles are too embarrassed to contact us?

One of the core values in Scandinavian general practice is the continuously developed strong relationship between GPs and patients. This relationship and subsequent knowledge about the patient give the GPs the possibility to provide tailored, individual, and balanced information and education to their patients about optimal healthcare seeking to reduce health anxiety, empower self-management where appropriate, and raise awareness of symptoms that warrant contact to the GP. These could obviously be symptoms indicating severe disease where prompt diagnostics could improve the prognosis. However, it may be equally important to focus on symptoms of benign but bothersome conditions where effective treatment or alleviation is possible, but many patients do not seek treatment [8, 10]. Also, the GP is the obvious healthcare professional to talk to his/her patients about their help-seeking behavior for acute problems, as the GP receives a summary of the out-of-hours contact. Knowing their patient makes it possible to provide guidance for similar episodes in the future. This guidance and patient education is an example of the “invisible work” we carry out each day; a consultation about a symptom is never just an evaluation a symptom, it often contains so much more.

The organization of healthcare systems is changing in these years of increased demands, lack of workforce, and limited resources. How we choose to organize primary care is central for our patients’ healthcare seeking possibilities and behavior [4]. The accessibility and availability of the GP are important for patients needing medical attention. For example, patients might feel a barrier for contacting their own GP outside telephone slots, as they have to call the acute telephone line where it for some patients can be difficult to evaluate whether their contact is acute enough. Research has shown a relation in the accessibility of daytime care and use of out-of-hours healthcare. GP practices with limited accessibility had more patients contacting the out-of-hours care than practices with good accessibility [11]. Geographical distances are important as well, even in a small country like Denmark, where participation in screening activities is associated with short distance to screening sites [12]. Patients who experienced a myocardial infarction and lived in small towns or rural areas and with a long distance to their GP had lower odds of having seen their GP prior to the incidents than those with short distance to their GP [13]. So, the chance for identification of patients at risk and initiating prevention might be lower in case of a long distance between the patient and the GP. This could be important knowledge to bear in mind when reforming the structure of the healthcare system in remote areas.

Although advertising is not necessary in general practice and we will probably never run out of work, it is important to continuously increase our knowledge about healthcare seeking to best guide and support patients in their decision-making and to prioritize those whose needs for healthcare are greatest.
